# Consequences of prenatal exposure to valproic acid in the socially monogamous prairie voles

**DOI:** 10.1038/s41598-019-39014-7

**Published:** 2019-02-21

**Authors:** L. Sailer, F. Duclot, Z. Wang, M. Kabbaj

**Affiliations:** 1 Program of Neuroscience, Department of Biomedical Sciences, Florida State University, Tallahassee, Florida 32306 USA; 2 Program of Neuroscience, Department of Psychology, Florida State University, Tallahassee, Florida 32306 USA

## Abstract

Environmental risk factors contribute to autism spectrum disorders (ASD) etiology. In particular, prenatal exposure to the highly teratogenic anticonvulsant valproic acid (VPA) significantly increases ASD prevalence. Although significant discoveries on the embryopathology of VPA have been reported, its effects on the ability to form enduring social attachment—characteristic of ASD but uncommonly displayed by rats and mice—remains unknown. We aimed to examine the effects of prenatal VPA exposure in the social, monogamous prairie voles (*Microtus ochrogaster*). Compared to prenatal vehicle-exposed controls, prenatal VPA-exposed prairie voles had lower body weight throughout postnatal development, engaged in fewer social affiliative behaviors in a familial context, exhibited less social interactions with novel conspecifics, and showed enhanced anxiety-like behavior. Along these behavioral deficits, prenatal VPA exposure downregulated prefrontal cortex vasopressin receptor (V1aR) and methyl CpG-binding protein 2 (MeCP2) mRNA expression, but did not alter spine density in adults. Remarkably, adult social bonding behaviors, such as partner preference formation and selective aggression, were not disrupted by prenatal VPA exposure. Collectively, these studies suggest that, in this animal model, VPA alters only certain behavioral domains such as sex-naive anxiety and affiliative behaviors, but does not alter other domains such as social bonding with opposite sex individuals.

## Introduction

The dynamic relationship between genetics, environmental factors, and epigenetic mechanisms renders vulnerability to potentially numerous neurodevelopmental disorders, such as schizophrenia and autism spectrum disorders (ASD)^1^. For instance, prenatal exposure to the histone deacetylase inhibitor (HDACi) sodium valproate (or valproic acid, VPA) has been linked to greater occurrence of ASD in humans^2–6^. A population-based study in Denmark observed a significant relationship between maternal use of VPA during pregnancy and increased risks of ASD in their children^7^. Histone deacetylases (HDACs) regulate the activity state of chromatin and repress gene expression through the removal of acetyl groups from the tail of core histones^8^. Akhtar *et al*. (2009) demonstrated that class I HDACi, such as VPA, form a developmental switch that modulates synapse maturation and functions by inhibiting HDAC1 and HDAC2 in immature and mature neurons. Inhibition of class I HDACs during early synaptic development causes a robust facilitation of excitatory synapse maturation and a modest increase in synapse numbers which may be associated with behavioral deficits observed in ASD^9^. Supporting evidence in rats and mice reveals that *in utero* exposure to VPA likewise induces ASD-like social behaviors, such as social interaction impairments, altered ultrasonic vocalizations, and repetitive auto-grooming^10,11^, as well as cellular and molecular deficits including alterations in glutamatergic neuronal differentiation^12^ and dendritic spine number in limbic regions^13^. Notably, the diverse effects of VPA are contingent upon the developmental period of administration^14^. For instance, the negative effects following prenatal VPA exposure do not appear during postnatal chronic treatment of HDAC inhibitors VPA and SAHA^15^. While studies with rats and mice have provided valuable insights into the effects of prenatal VPA exposure, few considered both sexes^12,14,16,17^. Interestingly, while a 4:1 male:female ratio is observed in the human ASD population^18^, the male:female ratio in children prenatally exposed to VPA who develop ASD is 1:1^19^. Most importantly, no studies have examined the effects of VPA in a relevant specie that exhibit strong social behaviors, which are deficient in ASD.

The prairie vole is an excellent animal model for understanding the neurobiology of prosocial behaviors and social cognitive deficits exhibited in psychiatric disorders. It is one of the rare animal species that exhibit social behaviors that recapitulate the complexity of some human social behaviors. The prairie vole is part of the 3% of mammalian species that are socially monogamous—forming long-lasting social attachments with their mating partners and displaying selective aggression toward intruders^20^—and provide sustained co-parental care for their offspring^21^. These behaviors involve neuropeptides and their receptors, such as oxytocin (OT) and vasopressin (AVP)^22,23^. Furthermore, the range of neural processes and behaviors modulated by OT and AVP exist in a sex-dependent manner in prairie voles^22,24^. For instance, sex-naive females have greater densities of OT receptor (OTR) binding but reduced densities of AVP receptor V1a (V1aR) binding in the medial prefrontal cortex (mPFC), a brain region implicated in complex cognitive and social behaviors, than sex-naïve males^25^. The functional role of mPFC-OT of female prairie voles has been identified pharmacologically, in which mPFC-OTR antagonism prevents mating-induced partner preference,^26^ whereas OT injections in the mPFC facilitate partner preference formation in the absence of mating^27^. Due to low mPFC-V1aR density in prairie voles and V1aR absence in cortical layers that receive dopaminergic inputs^25^, no pharmacological manipulations of V1aR have been implemented in prairie voles to examine its functional role in social attachment. However, validation of higher male-specific V1aR densities in brain regions that comprise the mesolimbic reward system indicates that male prairie voles are more sensitive to AVP than females. For example, mating triggers vasopressin release and activation of vasopressin V1aR receptors, while inhibition of vasopressin receptors prevents the formation of partner preference in male prairie voles^28,29^. Overexpression of V1aR, through genetic manipulation, in a promiscuous vole species results in the ability to form an exclusive partner preference^30^.

Importantly, evidence of irregular structure and function in the mPFC has been steadily reported in individuals with ASD^31^ and *in utero* VPA-exposed male rodents^13,32^. For instance, prenatal exposure to VPA enhances short- and long-term synaptic plasticity (*i.e*. paired-pulse facilitation and long-term potentiation, respectively) in the mPFC of male rats^33^. We therefore chose to probe, within the mPFC of male prairie voles, for factors known to regulate social bonding—OTR and V1aR—or synaptic plasticity genes involved in ASD pathology. In addition to alterations in gene expression, males with ASD^34,35^ and VPA-exposed male rats and mice^13,36,37^ exhibit hyperconnectivity in local circuits and hypoconnectivity between brain regions, which, along with alterations in spine density and morphology, are likely to underlie socio-cognitive impairments characteristic of this disorder. For instance, adolescent VPA-treated mice present with alterations in cortical neuron spine morphology (decreased number of filopodia and stubby spines and enhanced numbers of thin and mushroom spines, along with a decreased spine head size) concomitant with social interaction impairments^37^. Moreover, prenatal VPA exposure in male rats induces cortical spine density loss, with a shift from dendritic refraction to dendritic hypertrophy throughout development^13^.

Therefore, in this study, we aimed to validate the VPA model in male and female prairie voles to elucidate the molecular mechanisms by which social behaviors are perturbed, such as affiliation with cage-mates, interaction with novel conspecifics of the same sex, mating-induced partner preference, and selective aggression. We assessed how expression of genes important in the modulation of social behaviors are altered, and assessed how cortical spine density and morphology during adulthood is affected.

## Materials and Methods

### Animal Subjects

Male and female prairie voles (*M. ochrogaster*) were produced from laboratory-bred colonies at Florida State University, weaned on postnatal day (PND) 21 and housed with same-sex littermates. All animals received food and water *ad libitum* and were maintained at 20 °C on a 14:10 light-dark cycle. All procedures were conducted and approved by the Institutional Animal Care and Use Committee (IACUC) of Florida State University and were in accordance with the guidelines set forth by the National Institutes of Health.

### Prenatal exposure to valproic acid

**Figure 1:**
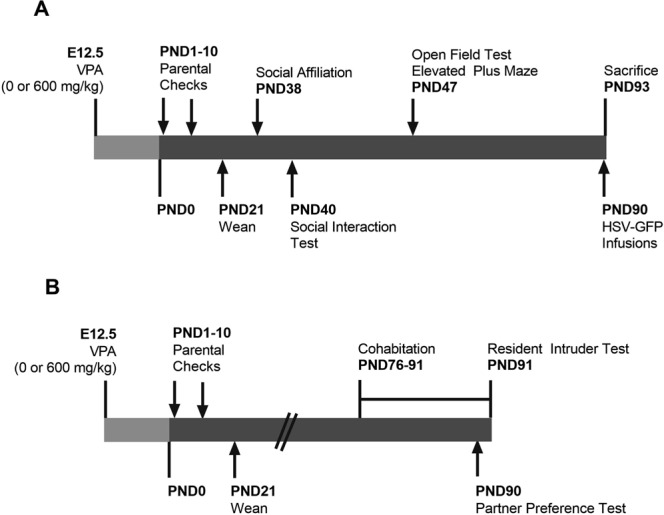
Experimental time lines of experiment 1 (**A**) and experiment 2 (**B**). E12.5, embryonic day 12.5; VPA, valproic acid; PND, postnatal day.

Adult (90 days old) sexually naïve female and male prairie voles were pair-housed and visually recorded for the first 3 days of cohabitation to confirm the day of mating—then considered as embryonic day 0 (E0). On gestation day 12.5 (Fig. 1), timed-pregnant female prairie voles received a single intraperitoneal (*i.p*.) dose of 600 mg/kg VPA (Sigma–Aldrich, St. Louis, MO, USA) or an equivalent volume of 0.9% saline (vehicle) and subsequently left undisturbed with their male partner and offspring until the time of pup weaning on PND21. The day and dose (600 mg/kg) of VPA was chosen as it is established to cause substantial social impairments in rats and mice^11,38^. Finally, the offspring of the time-mated dams were first separated by treatment and then by sex so that saline- or VPA-exposed male cagemates were pair-housed separately from pair-housed saline- or VPA-exposed female cagemates.

### Parental behavior spot checks

Maternal licking and grooming are critical for the development of social behaviors in rodents^39–41^. Furthermore, bi-parental care, a unique social behavior in prairie voles, is an important aspect for the development of social attachment^21,42^. In order to assess parental behaviors, spot checks were conducted twice daily from PND1–10 of the time-mated dams and sires, as described previously^42^. In the morning (0900) and late afternoon (1700), parental behaviors (*i.e*. nest occupancy, passive nursing, and active nursing) were thus scored by five spot checks conducted with 5-min interval for a total of 10 measures per day. All dam-sire pairs were included in the analysis.

### Measurement of body weight and weight gain

Body weights of each animal were recorded at different postnatal timepoints: on weaning (PND21), during adolescence (PND28 and PND35), and sexual maturity (PND90). Weight gain was calculated as: body weight of one timepoint (g) – body weight of PND21 (g).

## Experiment 1

In this experiment, we examined the effects of prenatal VPA exposure on social behaviors, prefrontal cortex gene expression of molecules previously implicated in ASD, and prefrontal cortex dendritic spine density^13,37^. To this end, male and female prairie voles prenatally-exposed to VPA or saline were weighed on PND21, 28, 35, and 90, and subjected to a series of behavioral tests on days PND38-47 to determine the effects of VPA on social affiliation, social interaction, anxiety-like behaviors, and locomotion during adolescence (Fig. 1A).

### Social affiliation

Adolescent affiliative behavior with a cage-mate was measured on PND38. Subjects were placed in a novel cage (12 × 28 × 16 cm) with their cage-mate for 30 minutes, during which the mean duration of affiliative behaviors (side-to-side contact), social interaction (anogenital sniffing, nose-to-nose sniffing, flank exploration, and social grooming), and non-social cage exploration were quantified by an observer blind to treatment condition using a computer data acquisition system (JWatcher_V1.0 Windows version with JRE 1.5.0, http://www.jwatcher.ucla.edu).

### Social interaction

The social interaction test, a modification of^43^, was performed on PND40. The three-chamber apparatus consisted of a neutral cage (12 × 28 × 16 cm) joined to two identical side cages by plastic tubes (7.5 × 16 cm) connected with sensors detecting chamber transition frequency and duration. Smaller, cubical wire cages were used to contain either the unfamiliar animal (UA) or toy in the side cages. The test subject was placed in the middle chamber with the tubes closed off and habituated for 10 minutes. At the end of habituation, the dividers were raised, allowing the test subject to move freely throughout all three chambers of the apparatus for a 10-minute test session. Location of the social side was alternated on consecutive sessions and the apparatus was cleaned with 70% ethanol (EtOH) between sessions. The duration and frequency of time spent sniffing the wired cages that contained either the UA or toy were quantified by an observer blind to treatment condition.

### Open Field Test

The open field test was performed as previously described^44^, to assess locomotor activity on PND47. Test subjects were placed in the center of a squared open field arena (56 cm L × 56 cm W × 20 cm H) and allowed to explore freely for 10 minutes. Their behavior was videotaped and EthoVision XT 8.5 (Noldus, Leesburg, VA, USA) was used post-testing to measure locomotion, scored as distance traveled (cm) during the 10 min. test. Visual cues were kept constant and the arena was cleaned with 70% EtOH in between sessions.

### Elevated Plus Maze Test

The testing apparatus for the elevated plus maze (EPM) consisted of two open arms (35 cm × 6.5 cm) and two closed arms (35 cm × 6.5 cm × 15 cm) that cross in their middle as previously described^45^. On PND47, subjects were placed in the center, facing a closed arm and behavior was videotaped for 5 minutes to assess anxiety-like behaviors. The latency to enter an open arm, as well as time spent and frequency of entries into each arm were scored by an experimenter blind to treatment conditions. The apparatus was cleaned with 70% EtOH between sessions.

### Tissue processing and RNA extractions

Male subjects that did not receive intra-mPFC HSV-GFP infusions were terminated by rapid decapitation on PND93, their brains rapidly extracted and frozen on dry ice before being stored at −80 °C. Sections anatomically matching Plates 9–13 in Paxinos and Watson^46^ were selected, tissue punches (1-mm diameter) were then taken bilaterally from the entire mPFC from 200 µm thick coronal sections, and stored at −80 °C until further processing for total RNA extraction using TRI-reagent according to the manufacturer’s protocol (Molecular Research Center) and as previously described^47^ Notably, one half of the animals per group was used for RNA extraction, whereas tissue from the other half of the males was used for chromatin immunoprecipitation.

### Semi-quantitative real-time PCR

Total RNA (350–500 ng) was reverse-transcribed with the cloned AMV First-Strand cDNA Synthesis Kit (Invitrogen, Thermo Fisher Scientific) to examine mRNA expression for *avpr1a*, methyl CpG-binding protein 2 (*mecp2*), *oxtr*, *nlgn1*, *bdnf*, *psd95*, *shank1*, *shank2*, and *shank3* by semi-quantitative real-time PCR in triplicates (See Table S1 for primer sequences). Primer specificity was verified by melt curve analysis. For each primer pair, amplified cDNA was normalized to nicotinamide adenine dinucleotide dehydrogenase (NADH), as described previously^47^, and presented as percentage of saline-exposed controls. All data was included in the analysis unless statistically defined as an outlier (>2 standard deviations from the mean).

### Chromatin immunoprecipitation

Phosphorylated cAMP response element-binding protein (pCREB) and MeCP2 binding at target genes promoters in the mPFC were analyzed by chromatin immunoprecipitation using the ChromaFlash High-Sensitivity ChIP Kit (Epigentek, Farmingdale, NY, USA) following the manufacturer’s instructions. Chromatin was sheared using a Bioruptor sonicator (Diagenode, Denville, NJ, USA) for 20 cycles (30 sec on, 30 sec off) with continuous cooling to fragments of 200–600 bp. Successful DNA immunoprecipitation was verified *a priori* with positive (RNA polymerase II) and negative (non-immune IgG) control antibodies. Immunoprecipitations for pCREB and MeCP2 were carried out with 0.10 µg of anti-pCREB (Millipore 06–519, Billerica, MA, USA) and 0.10 µg of anti-MeCP2 (Diagenode C15410052, Denville, NJ, USA). Immunoprecipitated DNA was analyzed in triplicates by real-time PCR with standard curves made from pooled DNA input samples to determine binding to the *avpr1a* and *mecp2* promoters. The primers were designed to amplify a 75-bp-long region located 216-bp upstream of the first exon coding for the prairie vole *avpr1a* gene (Genbank accession #AF069304) and an 87 bp-long region located 3,118 bp upstream of the first exon encoding the prairie vole *mecp2* gene (Genbank accession #NW004949275.1). As a positive control, pCREB binding to *bdnf*^48,49^ was analyzed (see Table S2 for primers sequences). Primer specificity was verified by melt curve analysis and each sample was normalized to the respective DNA input value and expressed as a percentage of Saline-treated voles. All data was included in the analysis unless statistically defined as an outlier (>2 standard deviations from the mean).

### Virus delivery, immunohistochemistry (IHC), spine density, and morphology measurements

At PND90, four males per group received bilateral stereotactic infusions of the HSV-GFP viral vector (p1005 + HSV plasmid expressing GFP under the control of cytomegalovirus promoter, prepared by Dr. Rachael Neve, MIT Viral Core Facility) into the medial prefrontal cortex (mPFC) as previously described^50^. Coordinates were as follows: +1.9 mm anterior, ±0.5 mm lateral, and −2.5 mm ventral relative to bregma. The viral vector was delivered at a rate of 0.1 µl/min using a 27-gauge syringe (Hamilton Laboratory Products, Reno, NV) for a total volume of 500 nL per side. Subjects were sacrificed and perfused 3 days after viral vector injection, and 50 µm thick coronal sections containing HSV-injection within the mPFC were processed for IHC as described previously^50^. Briefly, after blocking in 5% normal goat serum and 0.3% Triton X-100 for 1 hour, sections were incubated overnight with a chicken anti-GFP antibody (1:500; Abcam ab13970, Cambridge, MA) at 4 °C, and incubated with an Alexa Fluor 488–conjugated goat anti-chicken (1:1000; Life Technologies A11039, Grand Island, NY) for 3 hours. Labeled pyramidal neurons of layers III and V within the prelimbic region of the mPFC were imaged under 63× oil objective (Zeiss Plan Apochromat, numeric aperture = 1.40) of a Zeiss LSM880 confocal laser scanning microscope (Carl Zeiss, Germany) and the GFP tag was excited using an argon/krypton 488-nm laser line. For spine density and morphology analysis, Z-stacks of images were collected at 3x optical zoom and 0.39-µm step sizes using the Zen software (black edition, Carl Zeiss, Germany). Spine density and morphology were quantified using Neurolucida 360 and analyzed using Neurolucida Explorer and expressed as the number of spines per 10-µm dendritic segments, as previously described^50^. For each mPFC layer, 8 neurons per animal for each treatment were analyzed. One statistical outlier (>2 standard deviations from the mean) was removed from analysis. IHC did not work for a second animal and therefore its data was not included in that analysis.

## Experiment 2

In order to examine the effects of prenatal VPA exposure on partner preference formation and mating-induced aggression in adult male prairie voles, subjects were exposed to VPA at E12.5 and allowed to age to adulthood. At PND76, male prairie voles were pair-housed with ovariectomized females for 2 weeks—a paradigm known to reliably induce partner preference and selective aggression in prairie voles^51,52^—before a partner preference test on PND90 and resident-intruder testing at PND91 (Fig. 1B).

### Cohabitation and partner preference test

Immediately after cohabitation for 2 weeks (Fig. 1A), the partner preference test was conducted on PND90 as previously described in a three-chamber apparatus^47^. Briefly, the male subject was placed in the central cage connected at either side to two identical cages—one containing the female used for cohabitation (termed “partner”), and the other containing a stranger female—and allowed to freely explore for 3 hours. A trained experimenter blind to the treatment groups quantified the subject’s side-to-side contact duration with either the partner or stranger that included stationary huddling, sniffing, and grooming behaviors. Partner preference is defined as the subject spending significantly more time in side-by-side contact with the partner over the stranger. All animals in Experiment 2 were included in the analysis.

### Resident intruder test

On PND91, levels of selective aggression exhibited by VPA- and saline-exposed males was examined using the resident intruder test (RIT) for a period of 10 min as previously described^53^. After removing the female partner from the home cage of the male subject, a sex-naïve male intruder was introduced and the duration and frequency of aggressive behaviors (classified as lunges, bites, and chases) as well as latency to attack were scored^53^. All animals Experiment 2 were included in the analysis.

### Statistical analyses

All data are presented as the means ± SEM. Parental behaviors were analyzed by using a 3-way mixed ANOVA to compare the effects of sex, treatment, and postnatal day using SPSS Version 22 software (IBM Corp, Armonk, NY, USA). Body weight and weight gain were analyzed by using a 2-way mixed ANOVA to compare the effects of treatment and postnatal day. For comparisons of sex and treatment, data were analyzed by 2-way mixed ANOVA. For comparisons of sex, treatment, and stimulus preference, data was analyzed by using a 3-way mixed ANOVA. If any of these parent ANOVAs resulted in main effects or significant interactions (alpha set at 5%), Tukey’s post-hoc tests were performed to determine source of significance. All molecular data and experiment 2 data were analyzed using an unpaired two-tailed *t*-test after verification of normality. Additionally, partial Eta-squared (ηp^2^) and Cohen’s *d* are reported as measures of effect size for ANOVA and *t*-tests, respectively.

## Results

### VPA treatment to dams did not affect bi-parental behavior

**Figure 2:**
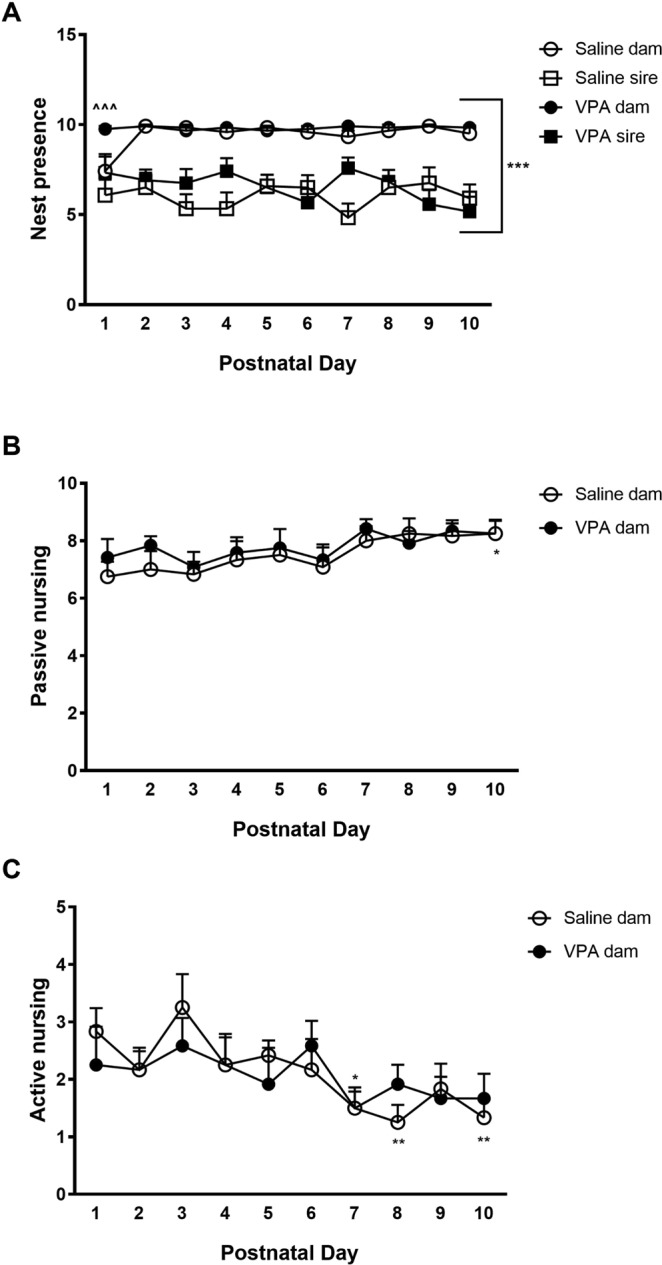
Evolution of bi-parental behaviors throughout postnatal days (PND) 1–10. Sires spent less time in the nest than dams (**A**). Dams engage in passive nursing (**B**) and active nursing (**C**) equivalently, regardless of treatment. Data are presented as mean ± SEM; n = 10 pairs/treatment, 2-way mixed ANOVA with Tukey’s post hoc test: ***p < 0.0001 dams vs sires; ^^^*p* < 0.001 VPA dam vs saline dam on PND1; *p < 0.05, **p < 0.01 vs saline dam on PND1.

We first assessed the effects of VPA treatment on bi-parental behaviors by conducting home-cage spot checks during the first ten postnatal days (PND1–10). Paternal presence in the nest was low when compared to maternal presence (Fig. 2A, Females vs Males, 3-way mixed ANOVA: F_1,44_ = 106.62, *p* < 0.0001, ηp^2^ = 0.71), but did not differ whether the partner dam was treated with saline or VPA (2-way mixed ANOVA, F_1,22_ = 0.73, *p* = 0.4005, ηp^2^ = 0.03). Interestingly, however, maternal nest occupancy was higher in VPA-treated dams on PND1 than in saline-injected dams (*p* < 0.0001), resulting in a different evolution of maternal presence in the nest over PND by VPA treatment (2-way mixed ANOVA, F_1,22_ = 7.35, *p* = 0.013, ηp^2^ = 0.251). Nevertheless, although passive and active nursing increased and decreased over PND, respectively (passive nursing: F_9,198_ = 2.56, *p* = 0.0084, ηp^2^ = 0.104 for PND; active nursing: F_9,198_ = 2.9, *p* = 0.003, ηp^2^ = 0.116 for PND; Fig. 2B), saline- and VPA-treated dams were undistinguishable (passive nursing: F_1,22_ = 0.44, *p* = 0.5123, ηp^2^ = 0.020 for treatment, F_9,198_ = 0.26, *p* = 0.9838, ηp^2^ = 0.012 for interaction; active nursing: F_1,22_ = 0.02, *p* = 0.880, ηp^2^ = 0.001 for treatment, F_9,198_ = 0.62, *p* = 0.7808, ηp^2^ = 0.027 for interaction), demonstrating that despite a lower maternal presence observed during the first PND, VPA injections do not affect bi-parental care in prairie voles.

It is important to note, however, that despite similar bi-parental care, VPA-exposed offspring exhibited lower body weight than saline-exposed controls from weaning (PND21) to PND90 (2-way mixed ANOVA, F_1,118_ = 16.32, *p* < 0.0001, ηp^2^ = 0.122 for treatment, F_3,354_ = 1112.65, *p* < 0.0001, ηp^2^ = 0.904 for time, and F_3,354_ = 1.479, *p* = 0.2199, ηp^2^ = 0.012 for the interaction, Fig. S1A). Nevertheless, VPA- and saline-exposed prairie voles exhibited a similar body weight gain from PND21 throughout the experiment (2-way mixed ANOVA, main effect of treatment: F_1,118_ = 1.28, *p* = 0.261, ηp^2^ = 0.011, main effect of time: F_1,118_ = 7.53, *p* = 0.007, ηp^2^ = 0.060, and for interaction: F_1,118_ = 0.14, *p* = 0.713, ηp^2^ = 0.001, Fig. S1B), suggesting an unaltered post-weaning development in VPA-exposed offspring.

### Exposure to VPA in utero alters adolescent social affiliation and social interaction

**Figure 3:**
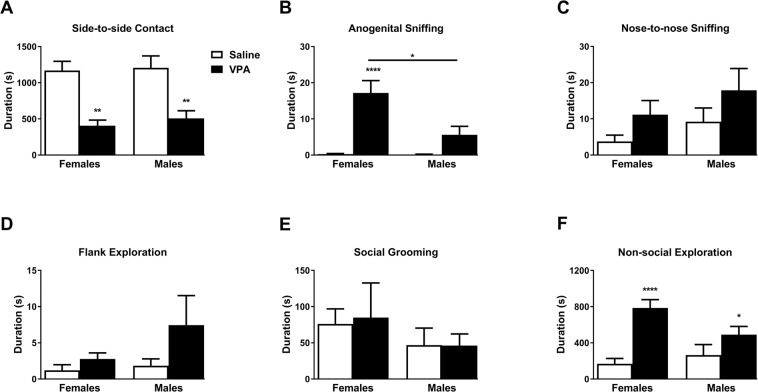
Adolescent social affiliation in response to exposure to VPA *in utero*. Duration of side-to-side contact with cage-mate (**A**), anogenital sniffing (**B**), nose-to-nose sniffing (**C**), flank exploration (**D**), social grooming (**E**), and non-social cage exploration (**F**) behaviors in prairie voles prenatally exposed to saline (white bars) or VPA (black bars). Data are presented as mean ± SEM; n = 8–9 animals/treatment, 2-way ANOVA with Tukey’s post hoc test.

In line with the known impairments in social behaviors following prenatal VPA exposure in rats and mice^14^, both male and female VPA-exposed offspring spent less time in side-to-side contact with a same-sex sibling than saline-exposed controls (F_1,29_ = 33.37, *p* < 0.0001, ηp^2^ = 0.535 for treatment, F_1,29_ = 0.29, *p* = 0.595, ηp^2^ = 0.010 for sex, and F_1,29_ = 0.065, *p* = 0.801, ηp^2^ = 0.002 for the interaction; Fig. 3A). Contrary to side-by-side contact, however, VPA-exposed prairie voles presented with a greater duration of anogenital sniffing than their saline-exposed counterparts in a sex-dependent manner, but not nose-to-nose sniffing (F_1,29_ = 3.77, *p* = 0.062, ηp^2^ = 0.115 for treatment, F_1,29_ = 2.16, *p* = 0.152, ηp^2^ = 0.069 for sex, and F_1,29_ = 0.02, *p* = 0.877, ηp^2^ = 0.001 for the interaction, Fig. 3C), flank exploration (F_1,29_ = 2.89, *p* = 0.100, ηp^2^ = 0.091 for treatment, F_1,29_ = 1.57, *p* = 0.220, ηp^2^ = 0.051 for sex, and F_1,29_ = 0.91, *p* = 0.348, ηp^2^ = 0.030 for the interaction, Fig. 3D), and social grooming (F_1,29_ = 0.02, *p* = 0.891, ηp^2^ = 0.001 for treatment, F_1,29_ = 1.33, *p* = 0.258, ηp^2^ = 0.044 for sex, and F_1,29_ = 0.02, *p* = 0.876, ηp^2^ = 0.001 for the interaction, Fig. 3E) in both females and males. Indeed, while prenatal VPA exposure led to higher duration of anogenital sniffing than saline-exposed controls overall (F_1,29_ = 30.63, *p* < 0.0001, ηp^2^ = 0.514 for treatment, Fig. 3B), this effect was more pronounced in females than males (F_1,29_ = 8.37, *p* = 0.0072, ηp^2^ = 0.224 for sex, and F_1,29_ = 7.98, *p* = 0.01, ηp^2^ = 0.216 for the interaction, Fig. 3B) and was reflected by higher duration of anogenital sniffing in VPA-exposed females than males (*p* = 0.002). Although similar effects on non-social exploration were observed, as VPA-exposed prairie voles exhibited higher durations than their saline-exposed counterparts overall (F_1,29_ = 19.99, *p* < 0.0001, ηp^2^ = 0.408 for treatment, Fig. 3F), sex differences were less pronounced. Indeed, despite a significant interaction with treatment (F_1,29_ = 4.32, *p* = 0.0467, ηp^2^ = 0.130 for sex:treatment interaction, and F_1,29_ = 1.14, *p* = 0.295, ηp^2^ = 0.038 for sex), VPA-exposed males and females spent a similar amount of time in non-social exploration (*p* = 0.149). Altogether, these observations indicate that prenatal VPA exposure reduces the duration of affiliative behaviors (*i.e*. side-to-side contact) in both male and female prairie voles, along with a coherent increase in non-affiliative (*i.e*. anogenital sniffing and non-social behaviors) that, interestingly, is more pronounced in females than males.

**Figure 4:**
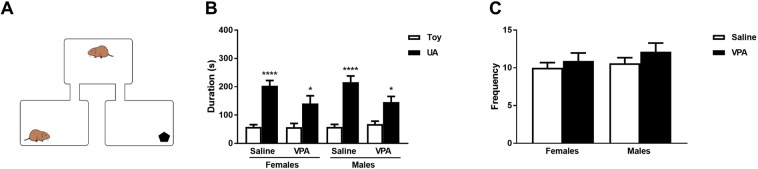
Prenatal VPA exposure reduces social interaction in females and males on PND40. (**A**) Representative image of social interaction test. (**B**) Time spent in contact with a novel toy or a same-sex unfamiliar animal (UA). (**C**) Total number of cage transitions (entry into toy cage + entry into UA cage), does not differ between VPA- and saline-exposed females (p = 0.881) and between VPA- and saline-exposed males (p = 0.676). Data are presented as mean ± SEM; n = 25–35 animals/treatment; mixed model 3-way ANOVA with Tukey’s post hoc test: *p < 0.05 and ****p < 0.0001 *vs*. toy; VPA: valproic acid.

Following examination of affiliative behaviors, social interaction with a novel same-sex conspecific and a toy was assessed on PND40 (Fig. 4A). Regardless of sex and treatment, all animals preferred to investigate wired cage containing an unfamiliar animal (UA) versus a toy during the social interaction test (mixed model 3-way ANOVA, stimulus main effect: F_1,93_ = 72.22, *p* < 0.001, ηp^2^ = 0.437, Fig. 4B). Yet VPA-exposed females and males significantly spent less time with the UA when compared to saline-treated females and males (mixed model 3-way ANOVA, stimulus:treatment interaction F_1,93_ = 5.64, *p* = 0.02, ηp^2^ = 0.057; stimulus:sex interaction: F_1,93_ = 0.98, *p* = 0.324, ηp^2^ = 0.010). VPA did not affect global investigative and exploratory behaviors as indicated by total cage transitions (Fig. 4C, 2-way ANOVA, F_1,116_ = 1.76, *p* = 0.187, ηp^2^ = 1.487 for treatment, F_1,116_ = 0.99, *p* = 0.321, ηp^2^ = 0.837 for sex, and F_1,116_ = 0.12, *p* = 0.734, ηp^2^ = 0.097 for the interaction). These data thus demonstrate that prenatal VPA exposure decreases social interaction with novel conspecifics during adolescence in both females and males.

**Figure 5:**
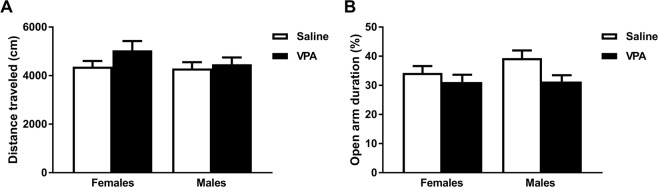
Adolescent anxiety-like behavior on PND47, but not locomotion, is altered by prenatal VPA exposure. Percentage of total time spent in the open arm in the EPM during a period of 5 min (**A**). Distance moved (cm/s) in an open field arena during a period of 10 min (**B**). Data are presented as mean ± SEM; n = 25–35 animals/treatment; 2-way ANOVA: *p < 0.05 main effect of treatment.

As hyperactivity and increased anxiety is often observed in individuals with ASD^54^ and in VPA-exposed rats and mice^14,55^, we tested whether VPA-exposed subjects at PND47 exhibit high locomotion on the OF (Fig. 5A) and anxiety-like behaviors on the EPM (Fig. 5B). Locomotion remained unaffected by sex or VPA (2-way ANOVA, F_1,117_ = 2.08, *p* = 0.152 for treatment, F_1,117_ = 1.19, *p* = 0.277 for sex, and F_1,117_ = 0.70, *p* = 0.1036 for the interaction). A main effect of treatment (2-way ANOVA, F_1,117_ = 5.21, *p* = 0.0243, ηp^2^ = 0.043), without interaction with sex (F_1,117_ = 0.99, *p* = 0.3206, ηp^2^ = 0.008) was observed in the percentage of time spent in the open arms. These data suggest that prenatal VPA exposure does not induce hyperactivity but increases anxiety levels in both the male and female offspring.

### Adult molecular- and plasticity-related alterations in response to prenatal VPA exposure

**Figure 6:**
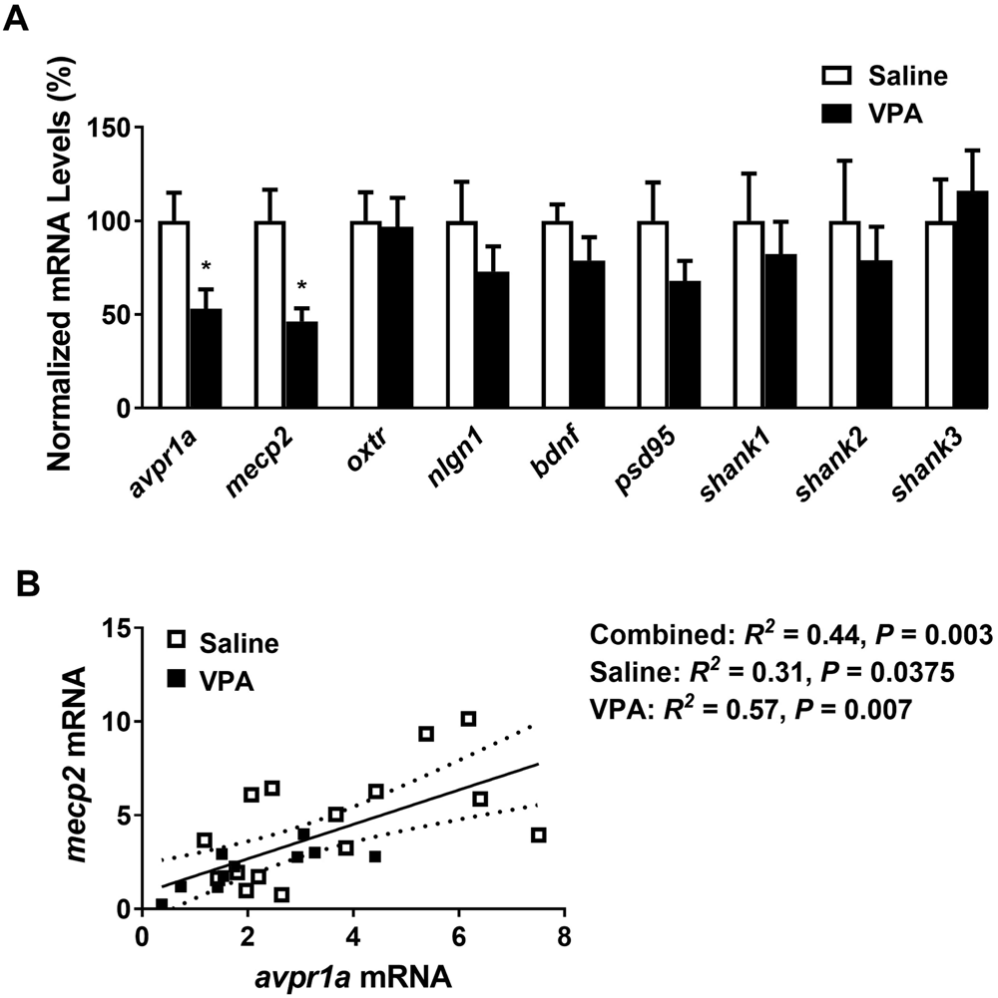
Expression of *avpr1a* and *mecp2* mRNA in the medial prefrontal cortex (mPFC) was downregulated by prenatal VPA treatment, but not *oxtr, nlgn1, bdnf, psd95, shank1, shank2*, and *shank3* (**A**). *Avpr1a* and *mecp2* mRNA in saline- and VPA-treated voles were positively correlated (**B**). Data are presented as mean ± SEM; n = 6–8 animals/treatment; two-tailed unpaired *t*-test: *p < 0.05 vs Saline. Dotted lines: SEM for a 95% confidence interval.

Although social and anxiety-like behaviors are similarly impaired in male and female VPA-exposed rats and mice^14,56^, the effects of VPA on mPFC synaptic gene expression and dendritic spine density have been reported primarily in males. This might be due to evidence of *SHANK1* deletions being exclusively associated with ASD in higher functioning men^57^ and men exhibiting significantly higher synaptic density than women in cortical layers I-VI, that suffer from temporal lobe epilepsy^58^, Prenatal VPA exposure induces a male-specific attenuation of MeCp2 protein expression in the mPFC of male rats and gender-isolated neural progenitor cells^16^. Taking these findings into consideration, we chose to examine molecular and cellular alterations in VPA-exposed male prairie voles. Prenatal exposure to VPA reduced *avpr1a* (two-tailed unpaired *t*-test, *t*_24_ = 2.481, *p* = 0.0205, *d* = 0.99) and *mecp2* (*t*_23_ = 2.704, *p* = 0.0127, *d* = 1.14) mRNA expression in the mPFC of male prairie voles, but not *oxtr*, *nlgn1*, *bdnf*, *psd95*, *shank1*, *shank2*, or *shank3* mRNA levels (Fig. 6A). Interestingly, a positive correlation between V1aR and MeCP2 mRNA in saline- and VPA-treated voles was observed in both experimental groups (Fig. 6B).

**Figure 7:**
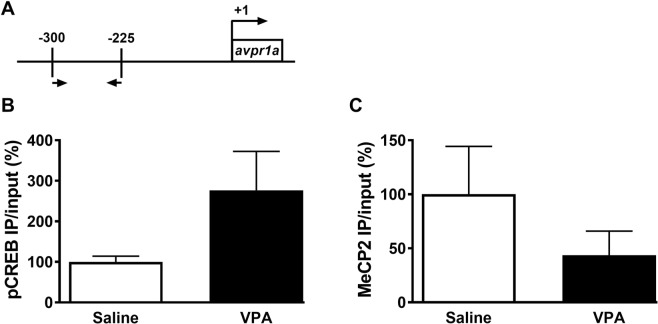
Chromatin immunoprecipitation (ChIP) was used to confirm phospho-CREB (pCREB) and MeCP2 predicted occupancy at the avpr1a promoter region. (**A**) Schematic representation the avpr1a promoter region where the genomic primers were designed (arrows) and their position relative to the transcription start site (+1 site). Prenatal VPA treatment does not alter pCREB (**B**) or MeCP2 (**C**) binding to the *avpr1a* promoter during adulthood. Data was normalized by the respective INPUT value, expressed as percentage of Saline-treated voles and presented as mean ± SEM; n = 5–6 animals/treatment.

Given that MeCP2 is a transcription factor, this positive association might suggest that MeCP2 mediates the down-regulation of *avpr1a*, as recruitment of CREB by MeCP2 at gene promoters results in transcriptional activation^59^. Alternatively, MeCP2 down-regulation could result from signaling events downstream of V1aR, involving for instance the transcription factor CREB and its active phosphorylated form. In order to test these two hypotheses, we next examined the binding of phospho-CREB and MeCP2 to the *avpr1a* promoter or phospho-CREB to the *mecp2* promoter in the mPFC of males, and whether binding was altered following prenatal VPA exposure. Opposing our first hypothesis of MeCP2’s role in mediating the down-regulation of *avpr1a* via recruitment of CREB (Fig. 7A), exposure to VPA does not alter the binding of either phospho-CREB (two-tailed unpaired *t*-test, *t*_9_ = 1.655 *p* = 0.1323, *d* = 1.05) or MeCP2 (*t*_8_ = 1.259, *p* = 0.2435, *d* = 0.77) to the *avpr1a* promoter in the mPFC, despite a high inter-individual variability (Fig. 7B,C). Interestingly, even though primers specificity at the *mecp2* promoter was successfully verified at two different loci using input DNA (Fig. S2A–H), no specific amplification of phospho-CREB immunoprecipitated DNA could be achieved at the *mecp2* promoter (Fig. S2A–S2H). In light of the successful amplification of the same samples at a positive control locus (*bdnf*, Fig. S2I,J), this suggests that phospho-CREB does not bind the *mecp2* promoter in our samples. In this context, the positive association between V1aR and MeCP2 mRNA levels is likely indirect, maybe through another common regulator of V1aR and MeCP2.

**Figure 8:**
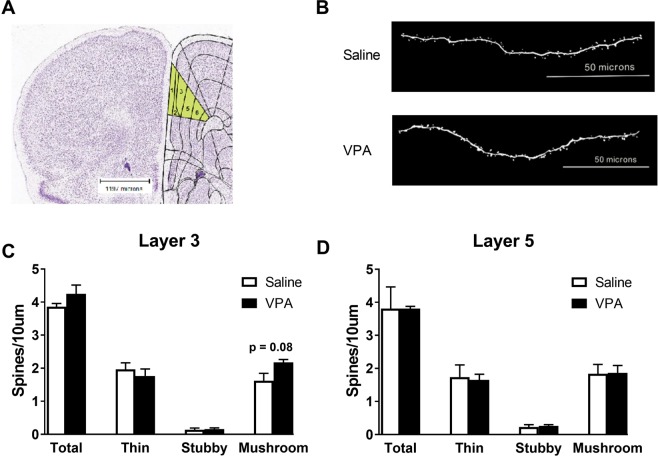
Influence of prenatal VPA exposure in spine density and spine morphology. Representative coronal section through the prefrontal cortex, the prelimbic cortex is highlighted in yellow with delineated pyramidal neuron layers, Image Credit: Allen Institute (**A**). Representative dendritic segments of pyramidal neurons from layer III using a 63x oil immersed objective (**B**). Total, thin, stubby, and mushroom spine density of pyramidal neurons of the layer III (**C**) and layer V (**D**) in the prelimbic cortex. Prenatal VPA treatment did not affect total spine density or spine morphology. Data are presented as mean ± SEM; 8 neurons/animal and n = 3.

Lastly in experiment 1, we analyzed dendritic spine density and morphology in pyramidal layers III and V neurons within the prelimbic region of the mPFC of adult male prairie voles prenatally exposed to VPA (Fig. 8A,B). In all measurements, saline and VPA-exposed males were undistinguishable (Fig. 8C,D), suggesting that behavioral alterations subsequent to prenatal VPA exposure are not associated with altered synaptic plasticity in the mPFC of adult prairie voles.

### Exposure to VPA in utero does not alter partner preference formation or selective aggression

**Figure 9:**
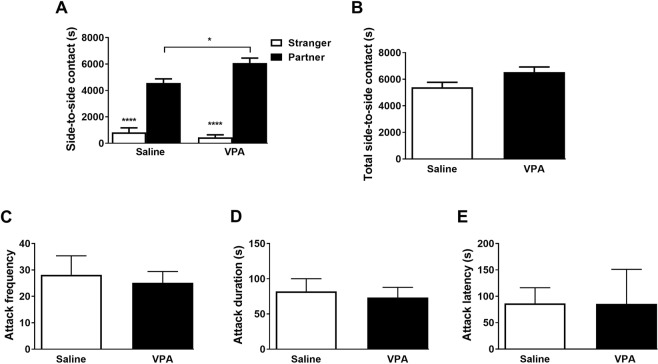
Prenatal VPA exposure does not prevent formation of social bond. (**A**) Saline- and VPA-treated males display a partner preference on PND90. (**B**) Total time spent interacting (stranger + partner). (**C**) Saline- and VPA-treated males display similar attack frequency towards unfamiliar male conspecific during the resident intruder test on PND91, as well as attack duration (**D**) and attack latency (**E**). *p < 0.05, **p < 0.01, ****p < 0.0001 *vs*. saline group. n = 9–12 animals/treatment.

In light of the deficits in social affiliation and social novelty observed above, we next investigated whether VPA-exposed prairie voles would retain their ability to form lasting social attachment, a pair-bond. To this end male prairie voles, prenatally exposed to either saline or VPA, were placed in cohabitation with a female in the presence of mating for two weeks and then tested for partner preference and selective aggression (Fig. 1B). Surprisingly, while both saline- and VPA-exposed males spent more time in side-to-side contact with the partner than with the stranger (2-way mixed ANOVA, F_1,19_ = 165.42, *p* < 0.0001, ηp^2^ = 0.897 for stimulus, F_1,19_ = 4.28, *p* = 0.052, ηp^2^ = 0.184 for treatment), VPA-exposed males spent more time huddling with their partner—but not their stranger—than saline-exposed males did (F_1,19_ = 6.64, *p* = 0.018, ηp^2^ = 0.259, Fig. 9A). Furthermore, the total time spent in side-to-side contact with either stimuli (partner + stranger) was similar in both groups (two-tailed unpaired t-test, *t*_19_ = 2.069, *p* = 0.052, *d* = 0.92, Fig. 9B), which rules out an interaction of prenatal VPA exposure with overall opposite-sex social interactions in this test. Altogether, these observations suggest that VPA-exposed males showed higher partner preference than saline-exposed controls. After 24 hours of respite, however, saline- and VPA-exposed males showed similar levels of selective aggression in the resident intruder test as measured by the frequency (two-tailed unpaired *t*-test, *t*_19_ = 0.335, *p* = 0.7411, *d* = 0.15) and duration (*t*_19_ = 0.357, *p* = 0.7249, *d* = 0.16) of aggressive behaviors, as well as the latency to attack (*t*_19_ = 0.008, *p* = 0.9934, *d* = 0.003) (Fig. 9C–E).

## Discussion

In the socially monogamous prairie voles, prenatal VPA exposure leads to behavioral and molecular alterations. Indeed, although VPA-treatment to the dam did not affect parental behaviors, the VPA-exposed adolescent female and male offspring showed reduced social affiliation and higher levels of non-affiliative behaviors with a same-sex sibling. Furthermore, VPA-exposed young female and male adults exhibit lower interaction with novel same-sex conspecifics and elevated anxiety levels than saline-exposed controls. These behavioral alterations are accompanied by a down-regulation of cortical V1aR and MeCP2 mRNA in adult males. Other synaptic and neuronal plasticity-related genes remained unaffected, as well as dendritic spine density and morphology in the mPFC of VPA-exposed male prairie voles. Surprisingly, despite deficits in social affiliations and interactions with a same-sex conspecific, VPA-exposed male prairie voles are still able to display partner preference and selective aggression. Altogether, these data thus indicate that prenatal VPA exposure in prairie voles induces a complex pattern of ASD-like molecular alterations and social deficits.

The attenuation of same-sex social affiliations and interactions observed following prenatal VPA exposure are, interestingly, accompanied by an increase in non-affiliative anogenital sniffing behaviors and occur in the absence of general locomotion impairments. It is important to note, however, that VPA-exposed animals also exhibited elevated anxiety levels, and thus conceivably explaining reduced social interactions with unfamiliar targets. Nevertheless, the observation in the same animals of substantial social deficits in a familiar context during affiliation with a same-sex sibling suggests that the effects of prenatal VPA exposure on social behaviors are not the sole result of an enhanced anxiety towards novel stimuli, but rather reflect more general impairments in social abilities between same-sex conspecifics.

Despite such general nature, the consequences of prenatal VPA exposure in adolescent prairie voles do not differ substantially between sexes. Indeed, the increase in same-sex non-affiliative behaviors was more pronounced in females than males. Whereas an equivalent male:female ratio is associated with ASD development in VPA-exposed children^19^, most rodent studies report the effects of prenatal VPA exposure on male offspring only^13,15^ or omit stating the sex^60,61^, and thus might lead readers to misinterpret that prenatal VPA exposure affects rodents exclusively in a male-specific manner. Of the few studies that examine sex differences in the induction of ASD-like alterations by prenatal VPA exposure^12,16^, our data is in line with VPA-exposed female mice investigating age-matched conspecifics more than VPA-exposed males, whilst exhibiting comparable behavioral responses in locomotion (in the open field) and anxiety levels (in the elevated plus maze)^14,56^.

Lastly, the origin of the complex profile of social behavior impairments triggered by prenatal VPA exposure remains unclear^62,63^. Interestingly, one contributing factor could be of environmental nature, through a direct effect of VPA on the dam’s maternal care towards the pups. This is especially intriguing in light of the positive association parental care and the offspring’s social behaviors during adolescence^64^. Following VPA treatment, however, we observed indistinct levels of parental care from PND1–10. We can thus rule out the possibility that social impairments in adolescent prairie voles result from different levels of parental care. Yet it is interesting to note that, comparably to VPA-exposed rats and *Mecp2*-null mice^65,66^, VPA-exposed offspring exhibited a marked lower body weight throughout postnatal development. In line with the timing of VPA exposure (E12.5) as a critical period for the induction of altered ASD-like behaviors and its known effects of neural tube closure in rats^67,68^, these findings point towards neurodevelopmental alterations rather than consequences of impaired parental behaviors.

Associated with Rett syndrome, MeCP2 expression is reduced in VPA-exposed mice^16^. Interestingly, evidence of MeCP2′s duality as a transcriptional repressor and activator^69^ opens the possibility of a direct transcriptional control of *avpr1a* by MeCP2 in our study, as supported by the down-regulation of both mPFC V1aR and MeCP2 mRNA expression in VPA-exposed male prairie voles. Nevertheless, pCREB and MeCP2 binding to the *avpr1a* promoter did not differ between VPA-exposed and saline-exposed groups, whereas pCREB binding at the *mecp2* promoter was undetectable. As a result, the positive association between V1aR and MeCP2 mRNA levels is likely indirect or CREB-independent. It is important to note, however, that these measures were characterized by high inter-individual variability that could therefore mask subtle differences. Despite the identification of *oxtr* as an ASD genetic risk factor^70^, mPFC OTR mRNA levels did not differ between saline- and VPA-exposed prairie voles. In agreement with our study, 20 mg/kg, but not 100 mg/kg, chronically VPA-exposed rats display reduced OTR binding in the amygdala but not in other limbic regions^71^. Thus our data supports this dose- and structure-specificity in the regulation of OTR expression following prenatal VPA exposure. Moreover, prenatal VPA exposure did not alter the expression of neuroplasticity genes *nlgn1*, *bdnf*, *psd95*, *shank1-3*, suggesting an unaltered neuronal plasticity in the mPFC of adult VPA-exposed prairie voles when compared to saline-exposed controls. Intriguingly, mutations of these synaptic molecules are associated with ASD pathogenesis in humans and ASD rodent models alike^72–74^. For example, MeCP2 down-regulation, with concurrent up-regulation of cortical PSD95 and Shanks 1–3, is linked to glutamatergic synapse development impairments in VPA-exposed rats during early postnatal life (PND14)^16^. Although Kim *et al*. (2016) focused on examining VPA-induced glutamatergic synapse alterations, the GABAergic neuronal marker glutamate decarboxylase (GAD) was down-regulated in the mPFC following prenatal VPA exposure without sex differences in rats but remained unchanged by MeCP2 siRNA-mediated knockdown in male- and female-derived neural progenitor cells^16^. Moreover, *in utero* exposure to VPA in mice does not alter the number of parvalbumin-immunoreactive GABAergic interneurons in the mPFC^32^. Notably, this mPFC hyper-synaptic function is normalized during adolescence (PND48) and then diminished in adult (PND120) VPA-exposed rats compared to saline-exposed controls^75^, suggesting that VPA-exposed neurons transition through a normal period between a hyper- to hypo-synaptic function. Conceivably, VPA-exposed prairie voles likewise undergo a homeostatic compensatory mechanism^76^ on PND90, explaining the evidence of unaltered mPFC neuroplasticity genes.

Accordingly, dendritic spine density and morphology in the adult mPFC were similar between VPA- and saline-exposed prairie voles. This finding can appear surprising in light of the spine pruning deficits and increased spine density observed in ASD patients^77^, or the reduced brain weight, cortical thickness, dendritic branching, spine density, and Nissl-positive cells in the PFC of mice and rats prenatally exposed to VPA^13,56,78^. For example, prenatal VPA exposure induces Nissl-positive cell loss in the mPFC of male and female mice^14,56^. Similarly, total and mushroom-type spine density in cortical neurons are reduced in Rett syndrome and related rodent models^79,80^. In MeCP2-null mice, however, while spine density is lower during early postnatal development, spine density is comparable to wildtypes during adolescence and adulthood when social deficits are well established, suggesting the enaction of a secondary compensatory mechanism^81^. Given the MeCP2 down-regulation observed in the mPFC without alterations in spines density at adulthood (PND90), we can thus speculate that prenatal VPA exposure in prairie voles may resemble Rett syndrome-like pattern of alterations in neuronal morphology. Nevertheless, future studies aimed at examining the role of MeCP2 on dendritic spine-subtype and total spine density throughout development in VPA-exposed male and female prairie voles are warranted.

Using the validated model of embryonic exposure to VPA (E12.5) in prairie voles, we observed a complex profile of impairments in same-sex social behaviors similar to those seen in rats and mice, thus confirming the validity of the prenatal exposure to VPA model in prairie voles. The socially monogamous nature of this specie, however, allows for between-sexes investigations, and especially, the formation of an enduring social attachment referred to as a pair-bond. Interestingly, despite impaired social affiliation with a same-sex sibling, VPA-exposed male prairie voles were capable of forming a pair-bond with a female, as reflected by the presence of partner preference and selective aggression. Moreover, VPA-exposed males huddled more with their partner than saline-exposed males, which would indicate a greater partner preference than controls. Nevertheless, prenatal VPA exposure reduces social interaction with novel conspecifics and increases anxiety levels, which could translate into a reduced motivation to interact with a stranger. Although no significant reduction in side-to-side contact with the stranger was detected, this could explain why VPA-exposed males show higher levels of partner preference but not selective aggression than saline-exposed controls. It is important to note, however, that this preserved ability to form an enduring pair-bond could also suggest that the effects of prenatal VPA exposure on social abilities in prairie voles are age-dependent, and thus no longer detected in adulthood, or can be reversed by social buffering through prolonged exposure to a partner^82^, a hypothesis worth examining in future studies. Our results nonetheless indicate that prenatal VPA exposure does not alter the ability of adult prairie voles to form and maintain a pair-bond, denoting a disconnection between social affiliative behaviors and the underlying processes of an enduring social attachment between sexes. Intriguingly, this is reminiscent of “high-functioning” ASD individuals, many of which continue to desire, initiate, and maintain sexual and romantic relationships despite experiencing difficulties with social communications and interactions^83,84^. In addition to recapitulating impairments in social interactions previously described in rats and mice, the prenatal VPA exposure in prairie voles thus allows for the specific investigation of affiliative behaviors within sexes and their disconnection with the formation of enduring social attachment between sexes.

### Conclusion and Future Directions

In summary, in order to assess how prenatal environment insults play a role in ASD pathology, this is the first study to confirm similarities between VPA-exposed male and female behaviors in distinct sex-naïve social contexts, to characterize mPFC molecular and cellular alterations in response to prenatal VPA exposure in males, and to show that mating-induced pair bonding in VPA-exposed males is unaltered. The results and contributions presented in this initial study should be considered in light of a number of limitations, given that females were only included in the sex-naïve behavior tests of experiment 1. Accordingly, future studies are warranted as studies on males cannot be generalized to females, since sex differences exist at all levels of biological organization, brain development, neuroanatomy, and neurochemistry; and sex differences in biology may lead to the susceptibility to the same behavioral deficits. Overall, three major queries remain to be addressed. Primarily is the need to examine the same mPFC molecular and cellular targets and test social bonding in saline- and VPA-exposed females. Second, the functional role of V1aR and MeCP2 in VPA-exposed prairie voles needs to be investigated, especially in various durations of cohabitation with a conspecific. We might expect to find sex differences, as OT and AVP regulate prairie vole social attachment in a sex-dependent manner^22,24^. Lastly, despite the down-regulation of mPFC *avpr1a* mRNA, VPA-exposed males were able to form a partner preference, thus we must assess other mesolimbic brain regions and their involvement in sex-specific regulation of sex-naïve and mating-induced social behaviors.

## Supplementary information

Supplementary material
