# Supplementary material for: Consequences of prenatal exposure to valproic acid in the socially monogamous prairie voles
Source: Sci Rep. 2019 Feb 21;9:2453. doi: 10.1038/s41598-019-39014-7 (PMC6385222; doi:10.1038/s41598-019-39014-7)
Supplement: Supplementary file 1 — Supplementary material [file 41598_2019_39014_MOESM1_ESM.pdf]

Consequences of prenatal exposure to valproic acid in the socially monogamous prairie voles

Author names

Sailer L[1], Duclot F[1], Wang Z[2], Kabbaj M[1]

Figure S1. Body weight is (***A***), but not weight gain (***B***), decreased by prenatal VPA exposure. Body weights were measured upon weaning (PND21), during adolescence (PND28 & PND35), and sexual maturity (PND90). Data pooled from males and females, presented as mean ± SEM; n = 25-35 animals/treatment; **p* < 0.05, 2-way mixed ANOVA with Tukey's post hoc test: **p* < 0.05, ****p* < 0.001.

Figure S2. Standard curves and melt peaks for specificity of mecp2 and bdnf qRT-PCRs against immunoprecipitated pCREB. The amplification efficiency (Eff%) for sequentially diluted input DNA at two mecp2 promoter loci was 101.1 and 106.0 ,respectively, with coefficients of determination (R^2) as 0.969 and 0.981, respectively (***A*** and ***E***). The uniform melt peaks indicate the same amplification products were formed with varying amounts of template (***B*** and ***F***). While the standard curves for detection of mecp2 from pCREB pull-down produces high primer set efficiencies (Eff% = 99.5, R^2 = 0.755 for ***C***, Eff% = 106.0, R^2 = 0.586 for ***G***), multiple melt peaks (***D*** and ***H***) are produced, indicating non-specific genomic DNA amplification. Amplified bdnf genomic DNA from pCREB pull-down produces high efficiency qPCR primer sets (Eff% = 105.1, R^2 = 0.986 for ***I***) and single melt peaks using varying amounts of DNA input (***J***), indicating specific amplification.

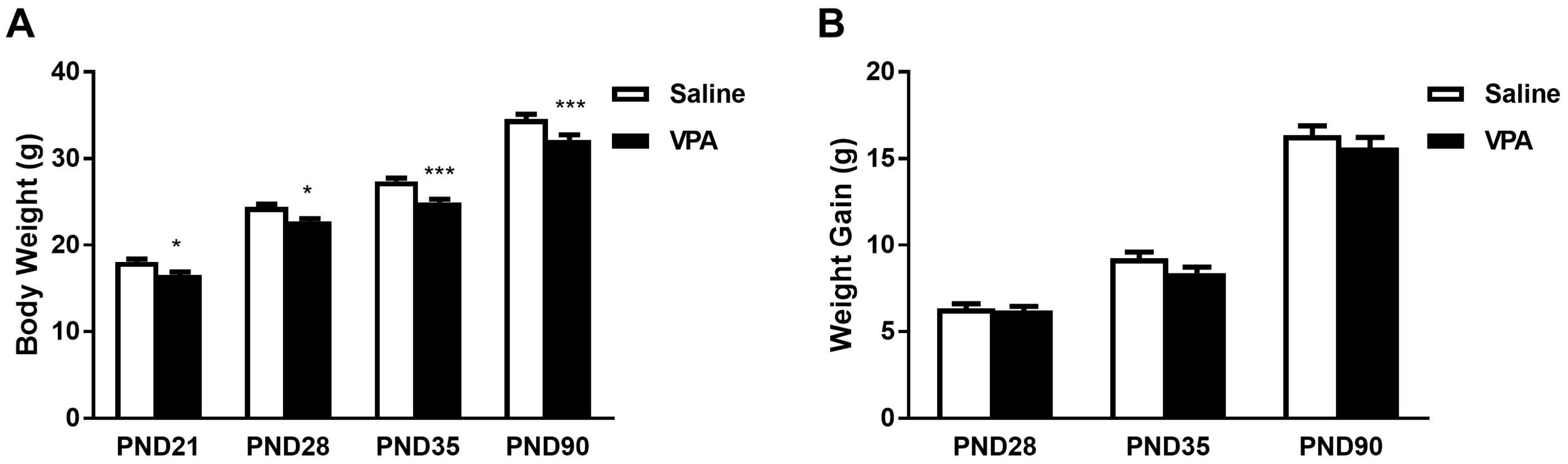
A
40
30
20
10
0
Body Weight (g)
*
*
***
***
PND21
PND28
PND35
PND90
Saline
VPA
B
20
15
10
5
0
Weight Gain (g)
PND28
PND35
PND90
Saline
VPA

A.

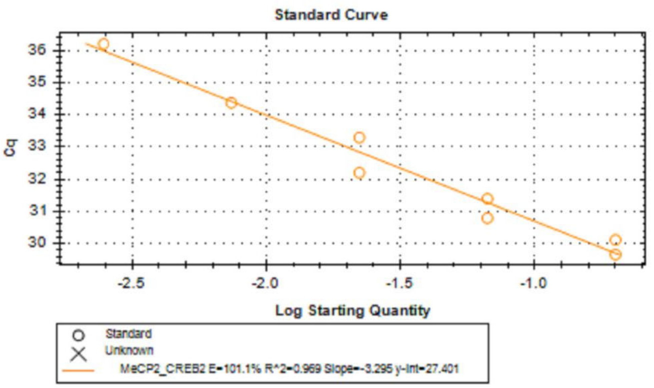
Standard Curve
Cq
Log Starting Quantity
Standard
Unknown
MeCP2_CREB2 E=101.1% R^2=0.969 Slope=-3.295 y-Int=27.401

B.

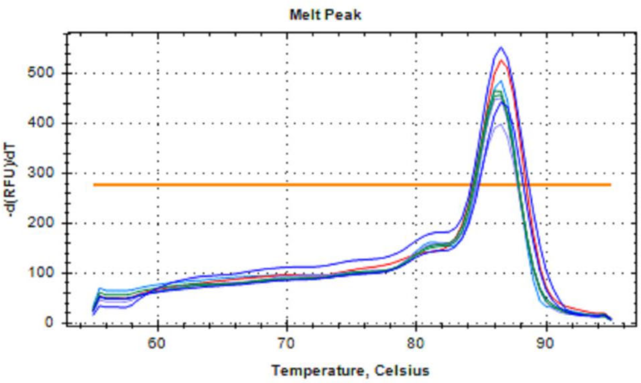
Melt Peak
-d(RFU)/dT
Temperature, Celsius

C.

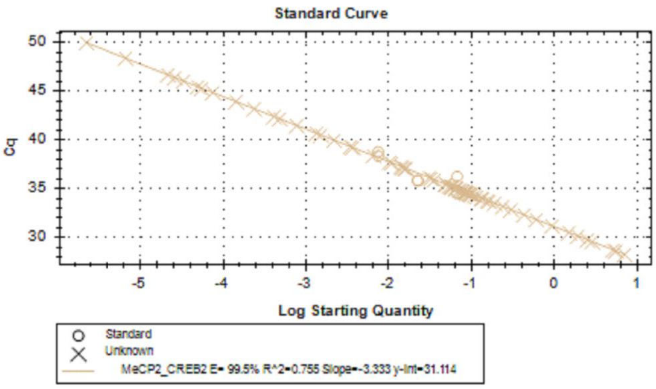
Standard Curve
Cq
Log Starting Quantity
Standard
Unknown
MeCP2_CREB2 E= 99.5% R^2=0.755 Slope=-3.333 y-Int=31.114

D.

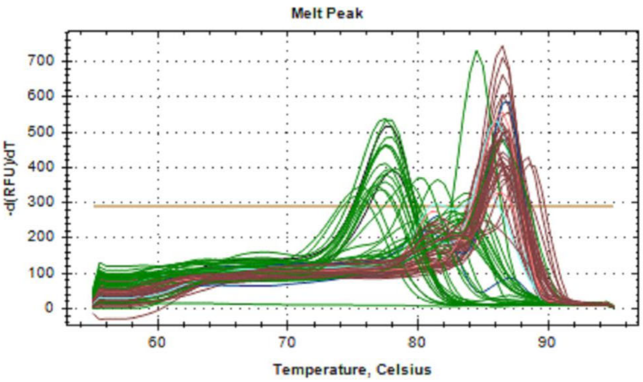
Melt Peak
-d(RFU)/dT
Temperature, Celsius

E.

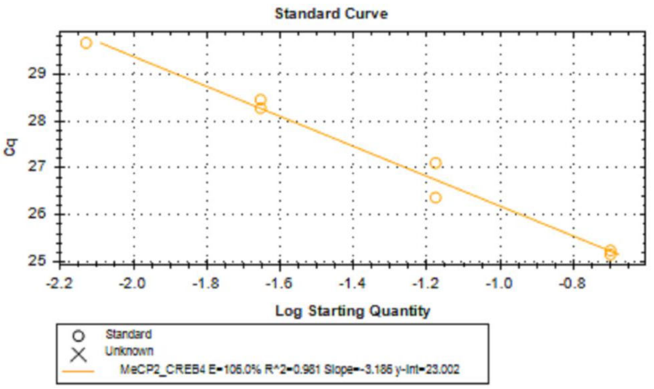
Standard Curve
Cq
Log Starting Quantity
Standard
Unknown
MeCP2_CREB4 E=106.0% R^2=0.981 Slope=-3.186 y-Int=23.002

F.

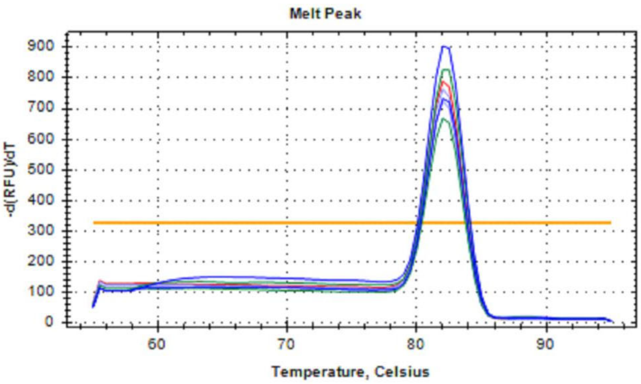
Melt Peak
-d(RFU)/dT
Temperature, Celsius

G.

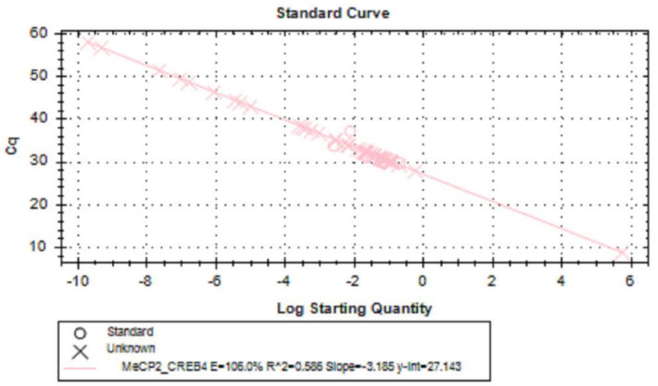
Standard Curve
Cq
Log Starting Quantity
Standard
Unknown
MeCP2_CREB4 E=106.0% R^2=0.586 Slope=-3.185 y-Int=27.143

H.

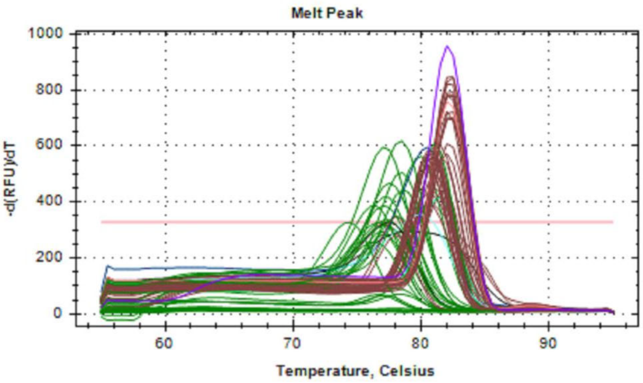
Melt Peak
-d(RFU)/dT
Temperature, Celsius

I.

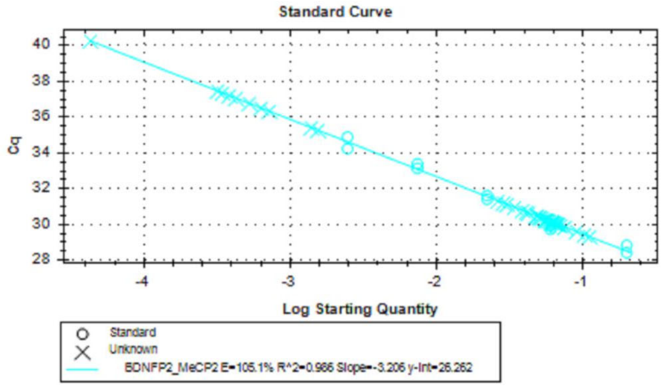
Standard Curve
Cq
Log Starting Quantity
Standard
Unknown
BDNFP2_MeCP2 E=105.1% R^2=0.986 Slope=-3.206 y-Int=26.262

J.

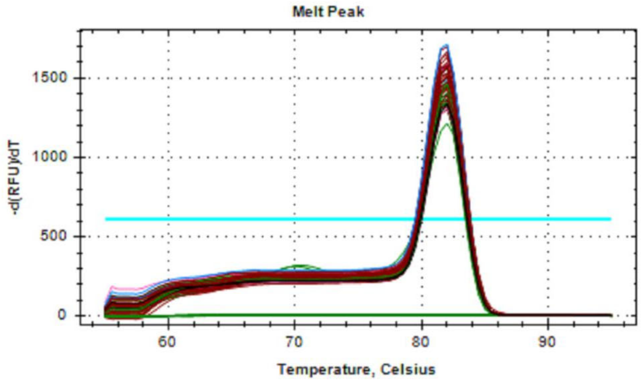
Melt Peak
-d(RFU)/dT
Temperature, Celsius

TABLES

Table S1. Primer sequences (5’-3’) used for amplification of target mRNA after Trizol extraction.

Target	Primer Sequence	
avpr1a	Forward	GAGGTGAACAATGGCACTAAAACC
	Reverse	CCAGATGTGGTAGCAGATGAAGC
mecp2	Forward	GAGGGAGAGCGCAAAGACAT
	Reverse	GCTAACTCTCTCGGTCACGG
oxtr	Forward	TCCAAGGCCAAAATCCGCACGG
	Reverse	GGCAGAAGCTTCCTTGGGCGC
nlgn1	Forward	CTTTCCAGCTGGGCTGTTAG
	Reverse	ATCGATCACAGGTCCAAAGG
bdnf	Forward	CCATAAGGACGCGGACTTGTAC
	Reverse	TTGGAGATGTGGTGGAGAGG
psd95	Forward	GGATATGAGTTGCAGGTGAACGG
	Reverse	TGAAGCCCAGACCTGAGTTACC
shank1	Forward	GCACAGACAGCCACCACGGA
	Reverse	GTCTTCAGAGAGCCTCTGCCGCT
shank2	Forward	CTGCAGAGAACGTGGCCATAGAATC
	Reverse	TCGTACACGGAGTTCACATCAGAGG
shank3	Forward	CAAGTCGTCCAGCCTCTCCATC
	Reverse	CCCACATCGAACTTGCTCCAAA
nadh	Forward	CTATTAATCCCCGCCTGACC
	Reverse	GGAGCTCGATTTGTTTCTGC

Table S2. Primer sequences (5'-3') used for amplification of target genomic DNA.

Target	Primer Sequence		Origin
avpr1a	Forward	CAGTCAGCGGCAGTAGACG	Own design
	Reverse	TGCTCCAGCCCCCTTTACAA	
*mecp2*_1	Forward	GGCTTTTACCACAGCCCTCT	Own design
	Reverse	CTCAAGCTGGCCGGGAG	
*mecp2*_2	Forward	AGATGTCACGCTCTTAGGGC	Own design
	Reverse	CAGTGCTAACCATCCGATCCA	
bdnf	Forward	CAGGTATTCTTTTCCTCGCTGT	[49]
	Reverse	CGCTCCAAAATCTGACTCTCTC	
